# First validation of myocardial flow reserve assessed by dynamic ^99m^Tc-sestamibi CZT-SPECT camera: head to head comparison with ^15^O-water PET and fractional flow reserve in patients with suspected coronary artery disease. The WATERDAY study

**DOI:** 10.1007/s00259-018-3958-7

**Published:** 2018-03-01

**Authors:** Denis Agostini, Vincent Roule, Catherine Nganoa, Nathaniel Roth, Raphael Baavour, Jean-Jacques Parienti, Farzin Beygui, Alain Manrique

**Affiliations:** 1 Department of Nuclear Medicine, CHU Caen, CHU Cote de Nacre, Caen, France; 2 Normandy University, EA 4650 Caen, France; 3 Department of Cardiology, CHU Caen, Caen, France; 4 Spectrum Dynamics Medical Ltd., Caesarea, Israel; 5 Department of Biometrics and Statistics, CHU Caen, Caen, France; 6 Cyceron PET Center, Caen, France

**Keywords:** Myocardial flow reserve, Myocardial blood flow, CZT camera, Dynamic SPECT, ^15^O–water PET, Fractional flow reserve, Coronary artery disease

## Abstract

**Purpose:**

We assessed the feasibility of myocardial blood flow (MBF) and flow reserve (MFR) estimation using dynamic SPECT with a novel CZT camera in patients with stable CAD, in comparison with ^15^O–water PET and fractional flow reserve (FFR).

**Methods:**

Thirty patients were prospectively included and underwent FFR measurements in the main coronary arteries (LAD, LCx, RCA). A stenosis ≥50% was considered obstructive and a FFR abnormal if ≤0.8. All patients underwent a dynamic rest/stress ^99m^Tc-sestamibi CZT-SPECT and ^15^O–water PET for MBF and MFR calculation. Net retention kinetic modeling was applied to SPECT data to estimate global uptake values, and MBF was derived using Leppo correction. Ischemia by PET and CZT-SPECT was considered present if MFR was lower than 2 and 2.1, respectively.

**Results:**

CZT-SPECT yielded higher stress and rest MBF compared to PET for global and LAD and LCx territories, but not in RCA territory. MFR was similar in global and each vessel territory for both modalities. The sensitivity, specificity, accuracy, positive and negative predictive value of CZT-SPECT were, respectively, 83.3, 95.8, 93.3, 100 and 85.7% for the detection of ischemia and 58.3, 84.6, 81.1, 36.8 and 93% for the detection of hemodynamically significant stenosis (FFR ≤ 0.8).

**Conclusions:**

Dynamic ^99m^Tc-sestamibi CZT-SPECT was technically feasible and provided similar MFR compared to ^15^O–water PET and high diagnostic value for detecting impaired MFR and abnormal FFR in patients with stable CAD.

## Introduction

D-SPECT, a high-sensitivity dedicated cardiac CZT-SPECT camera, allows dynamic acquisition of tomographic images suitable for in vivo assessment of radiotracer kinetics and opens up a new era for myocardial flow and flow reserve measurement using SPECT imaging [1–3].

Positron emission tomography (PET) using oxygen-15-labeled water (^15^O–water) is the gold standard for non-invasive assessment of myocardial perfusion, as ^15^O–water is freely diffusible with an extraction fraction independent of flow rate [4]. ^15^O–water PET has been validated in animal models [5, 6] and is highly reproducible in humans [7, 8]. Adding myocardial flow reserve (MFR) quantification with PET offers incremental diagnostic and prognostic information over conventional myocardial perfusion imaging [9].

Recently, fractional flow reserve (FFR) has emerged as a standard of care for clinical decision making in patients with ≥50% coronary artery stenosis who are candidates for percutaneous intervention [10, 11]. However, while FFR is a derived ratio of intra-coronary pressures, MFR integrates all hemodynamic determinants of myocardial perfusion at rest and during hyperemia, including the epicardial arteries and prearterioles. Cardiac oxygen PET is unfortunately limited to major academic labs and research centers, and therefore a SPECT alternative is an attractive option. Recent preliminary data demonstrated the feasibility of SPECT measurement of MFR; however, no quantitative estimates of MFR obtained with dynamic ^99m^Tc-sestamibi CZT-SPECT have been compared to invasive FFR and ^15^O–water PET in patients with suspected of coronary artery disease (CAD).

The aim of the prospective WATERDAY study was to validate the quantification of regional and global MBF and MFR obtained from dynamic ^99m^Tc-sestamibi imaging using a CZT-SPECT camera in patients with stable angina in comparison to ^15^O–water PET and FFR.

## Materials and methods

### Patient population

From November 2014 to November 2016, 109 consecutive, stable, symptomatic patients with positive exercise stress test (downsloping ST segment depression <1 mm) or perfusion imaging (reversible perfusion defect in at least 2 out of 17 myocardial segments) were referred for diagnostic invasive coronary angiography (ICA) and screened for possible eligibility in the WATERDAY trial. Information regarding patient age, gender, weight, medical history, risk factors (family history of CAD, hypertension, diabetes, smoking or hypercholesterolemia), medication, blood pressure, heart rate and symptoms were recorded. Among these patients, 45 were enrolled in the WATERDAY study (clinicaltrials.gov unique identifier NCT02278497) and underwent FFR measurement.

Inclusion criteria were: patients between 18 and 80 years old, with angiography-proven CAD, affiliated to a social security system, who have been informed of the study and gave informed consent. Exclusion criteria were: absence of ≥50% stenosis of at least one artery at ICA, absence of FFR measurements on the 3 main epicardial arteries, delay greater than 30 days between both PET and SPECT imaging and the ICA, patient with a recent history of acute coronary syndrome, patient with extra-cardiac disease whose prognosis can interfere with the treatment decision, pregnant or lactating women, allergy to angiographic contrast media, patient with renal failure with a modification of diet in renal disease (MDRD) clearance less than 60 ml/min, patient under guardianship, or unable to understand the purpose of the study.

**Fig. 1:**
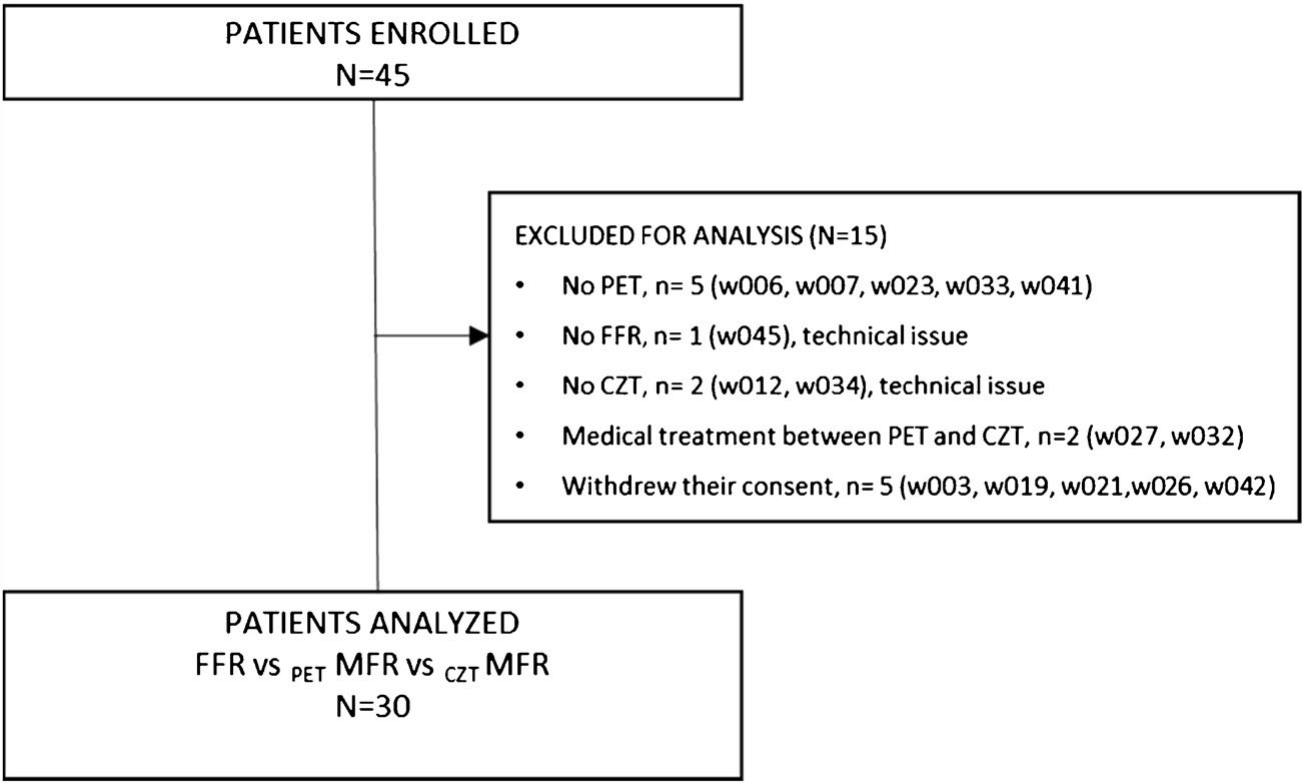
Flow chart of the WATERDAY study

Finally, only 30 out of 45 patients enrolled underwent both dynamic SPECT and PET within 30 days following ICA (Fig. 1). To avoid potential changes in MBF and MFR measurements, no revascularization procedure or medical therapy optimization was performed in any of the 30 analyzed patients between PET and SPECT imaging (Table 1). ICA/FFR identified 51 significant coronary artery lesions (Table 2).

**Table 1:** Patients’ characteristics

(*n* = 30)	Value
Male gender	21 (70%)
Mean age ± SD (y)	65 ± 7.1
CAD risk factors (n)	
BMI >30	8 (27%)
Diabetes mellitus	10 (33%)
Hypertension	20 (67%)
Dyslipidaemia	18 (60%)
Smoking history	17 (57%)
Family history	7 (23%)
>3 CAD risk factors	15 (50%)
Chest pain	15 (50%)
Medical therapy	
Clopidogrel	4 (13%)
Aspirin	30 (100%)
β-blockers	18 (60%)
ACE inhibitors or AT-II antagonist	17 (57%)
Calcium channel blockers	6 (20%)
Coumadine	2 (7%)
Statins	25 (83%)
Oral antidiabetic	12 (40%)
Long acting nitrates	6 (20%)
Diuretics	3 (10%)
Insulin	3 (10%)

**Table 2:** Imaging findings and angiography data in the overall patient population (*n* = 30)

	n (percentage)*
Positive exercise stress test	26 (87%)
Positive stress MPI	18 (60%)
1-vessel disease	14 (47%)
2-vessel disease	11 (37%)
3-vessel disease	5 (16%)
Coronary lesions LM	1 (3%)
LAD	20 (66%)
LCx-Mg	13 (43%)
RCA	11 (37%)
Other	
Diagonal artery	4 (13%)
Ramus intermedius artery	1 (3%)

MPI: myocardial perfusion imaging; LM: left main coronary artery, LAD: left anterior descending artery, LCx-Mg: circumflex and marginal arteries, RCA: right coronary artery

The study protocol was approved by the Regional Ethics Committee (CPP Nord-Ouest III, France), written informed consent was obtained from all the patients and the procedures were in accordance with the Declaration of Helsinki.

### Coronary angiography and FFR

Maximal hyperemia was obtained by intravenous (i.v.) infusion of adenosine [200 μg/kg/min for the left anterior descending artery (LAD) and left circumflex artery (LCx), and 100 μg/kg/min for the right coronary artery (RCA) [12]. FFR was measured using a sensor-tipped 0.014-in guide wire (PressureWire; St. Jude Medical, St. Paul, MN, USA) in all coronary arteries (LAD, LCx, and RCA) with a diameter of ≥2 mm, and for every stenosis ≥50% in a vessel segment ≥2 mm in diameter. FFR was calculated as the ratio of mean distal pressure divided by the mean proximal pressure. A pullback recording was performed and recorded for each measurement.

### PET imaging

#### Dynamic ^15^O–water PET acquisition

Subjects were instructed to fast for 4 h and abstain from caffeine, theophylline and cigarette smoking for 24 h. All acquisitions were performed using a GE Discovery VCT RX (GE Healthcare, Buc, France), and started with a low-dose transmission CT scan for attenuation correction (AC; helical coverage: 40 mm, rotation time: 0.5 s, pitch 0.516:1, table speed: 20.62 mm/rot, helical thickness: 3.75 mm, 120 kV and 10 mA, leading to a DLP of 15.35 mGy.cm and a radiation exposure of 0.21 mSv in all patients). A first i.v. hand injection of ^15^O–water (1.5 to 3 MBq/kg) was performed at rest simultaneously with the start of a 24-frame (14 × 5 sec, 3 × 10 sec, 3 × 20 sec and 4 × 30 sec) 3dimensional (3D) dynamic emission scan. After allowing the decay of oxygen-15 radioactivity, patients underwent a bolus injection of regadenoson (400 μg) followed by a second hand injection of ^15^O–water (1.5 to 3 MBq/kg) and dynamic PET acquisition.

#### PET image analysis

After Fourier rebinning, emission sinograms were corrected for random coincidences and dead time and then reconstructed with the PET manufacturer’s attenuation and scatter correction using filtered backprojection (Hanning filter, cut-off 8 mm). MBF was quantified using the Carimas 2.4 software (Turku PET Centre, Turku, Finland) [7]. After reorientation of the data to the standard short axis, the software automatically searches for ventricle borders and locates initial regions of interest (myocardium, left and right ventricles), which can be modified manually. Based on this segmentation, stress and rest MBF (mL/min/g) were calculated using a single compartment model [8] and computed for the whole left ventricle (LV) and for each coronary territory (LAD, LCx, RCA).

### CZT-SPECT imaging

### Dynamic ^99m^ Tc-sestamibi CZT-SPECT acquisition

The D-SPECT system is comprised of nine individual detector columns, which rotate independently and are able to focus on a fixed region of interest (ROI). Throughout the dynamic list mode acquisition, the detectors perform a continuous step and shoot a scanning pattern consisting of multiple sweeps forward and backwards. Each sweep includes 10 positions over approximately 3 s. Therefore, a frame of 30 s would contain information from 10 sweeps while a frame of 3 s would contain information from only a single sweep. Rest and stress dynamic images are acquired in list mode over 6 min.

**Fig. 2:**
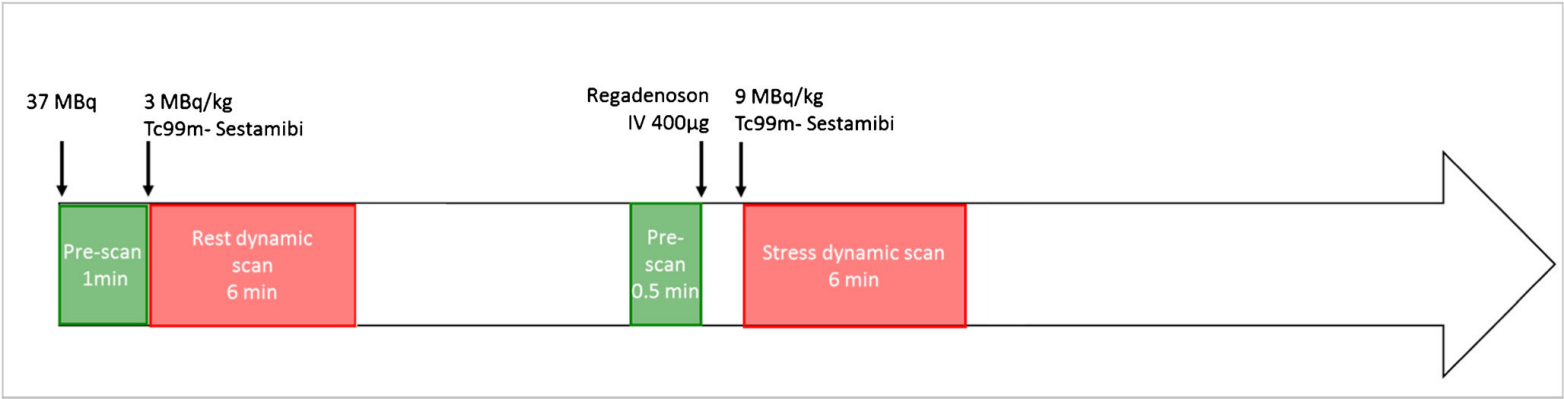
Dynamic CZT-SPECT imaging protocol

For rest imaging, an initial dose of approximately 37 MBq of ^99m^ Tc-sestamibi was used to position the patient’s heart within the field of view [13]. Three MBq/kg was then injected at a rate of 1–2 cm^3^/s using an automatic injector (Nemoto, Tokyo, Japan) and flushed by 30 mL of saline to ensure consistent delivery of a tight bolus. Regadenoson (400 μg) was then administered, followed by the injection of 9 MBq/kg of ^99m^Tc-sestamibi at peak hyperemia. Rest–stress dynamic acquisitions were completed within 75 min. Data were rebinned into 32 frames consisting of 21 × 3-sec, 1 × 9-sec, 1 × 15-sec, 1 × 21-sec, 1 × 27-sec and 7 × 30-sec frames. An ordered subset expectation maximization (OSEM) algorithm was used for image reconstruction with 4 iterations and 32 subsets. The procedure for dynamic CZT-SPECT imaging is summarized in Fig. 2.

#### Dynamic SPECT image analysis

Dynamic imaging data and corresponding perfusion information were analyzed using commercially available Corridor 4DM software (v2015, INVIA, Ann Arbor, MI, USA). LV endocardial and epicardial surfaces were algorithmically estimated from summed myocardial images beyond the 2-min mark. A midwall surface was divided into 460 polarmap sectors, where LV myocardial tissue time activity curves (TACs) were nearest-neighbor-sampled at the center of each sector across all time frames. Global and regional TACs (LAD, LCx, RCA) were averaged from the polarmap sector TACs. The MFR analysis used blood sampling by averaging a 3D box-shaped ROI within the LV/left atrium (LA) blood pool, localized in the LA in the short axis and centered at the basal valve plane along the long axis, across all time frames. The size of the ROI was two pixels wide in the short axis and 30 mm long in the long axis to sample both the LV and LA cavities [14]. A net retention model proposed by Jeffrey Leppo [15] and Yoshida [16] was used to calculate the retention rate R according to the following equation:

$$ R= MBF\times E=\frac{\frac{1}{PV\times \left({t}_3-{t}_2\right)}{\int}_{t_2}^{t_3}P(t)-{S}_m\times {C}_a(t) dt}{(CF){\int}_0^{t_1}{C}_a(t)-{S}_b\times P(t) dt} $$

In this equation, MBF is the myocardial blood flow, E is the extraction fraction, P(t) is the total myocardial tracer concentration or tissue TAC, C_a_(t) is the arterial concentration of the tracer or blood TAC and PV is the partial volume value. The correction factor for myocardial density CF was set to 1, S_m_ is the spillover from the blood pool activity to the myocardium estimated from compartmental analysis [17, 18], and S_b_ is the spillover from the myocardium to the blood pool activity which can be set to 0.0 assuming the spillover is negligible. Integration limit t_1_ denotes the end of the blood pool phase, typically at 1.5 min, while t_2_ and t_3_ denote integration limits of the average tissue activity, typically from 1.5 min to 2.5 min. The integration limits are adjusted to the peak of the blood TAC. According to Leppo [15], the uptake rate K1 was related to MBF using the following Renkin-Crone equation, where A = 0.874 and B = 0.443:

$$ K1= MBF\ast \left(1-A\ast {e}^{-\frac{B}{MBF}}\right) $$

### Statistical analysis

Continuous variables are expressed as mean ± SD and were compared using one-way ANOVA. Reproducibility for paired studies was examined by the Pearson correlation coefficient and Bland-Altman plot analysis. Abnormal global or regional stress MBF and MFR were defined using PET results as stress MBF < 2.5 ml/min/g and MFR < 2, respectively [19, 20]. The diagnostic value of CZT-SPECT for stress MBF impairment and abnormal MFR was assessed using receiver-operating characteristic (ROC) analysis. Concordance between categorical variables was assessed using a Kappa test. All tests were two-tailed and a *P* value ≤0.05 was considered statistically significant.

## Results

### Demographics

Figure 1 presents the flow chart of the study, and patients’ characteristics are summarized in Table 1. Regadenoson infusion was well-tolerated in all cases and hemodynamically [heart rate (HR) and blood pressure (BP)] responses did not change significantly during both modalities at rest (CZT-HR = 65.3 ± 11 vs. PET-HR: 65.71 ± 10 bpm; CZT-systolic BP = 118 ± 18 vs. PET = 120 ± 21 mmHg; diastolic CZT-BP = 66.5 ± 9 vs. PET = 63 ± 8 mmHg) and at stress (CZT-HR = 85.45 ± 13 vs. PET-HR = 84.6 ± 15 bpm; CZT systolic BP = 100 ± 20 vs. PET =102 ± 17 mmHg; diastolic CZT-BP = 65 ± 8 vs. 64.6 ± 6 mmHg; *P *= NS for all data). However, regadenoson induced significant increase of HR and decrease of SBP between rest and stress in both modalities (*P* < 0.05).

### Comparison of dynamic CZT SPECT vs. PET.

The inter-observer (DA, CN) reproducibility of MBF and MFR measurements was excellent (*r* = 0.92). The software (Corridor 4DM) offered the possibility to manually correct the positioning of the VOI in case of failure of the automatic processing and we applied it in two cases essentially by drawing VOIs in the LA.

Table 3 shows all myocardial blood flow and reserve quantification for the whole LV and for vessel territories assessed by CZT-SPECT and PET in 30 patients. As expected, pharmacological stress significantly increased MBF in both modalities. CZT-SPECT yielded higher stress and rest MBF compared to PET for global, and LAD and LCx, territories but not in RCA territory. Nevertheless, MFR was similar in global, and each vessel territory, for both modalities. No significant difference in MFR quantification was observed in each vessel territory between CZT or PET.

**Table 3:** Global and regional myocardial flow reserve estimates from dynamic CZT-SPECT and PET

	Stress MBF (ml/min/g)			Rest MBF (ml/min/g)			MFR		
	CZT	PET	*P* value	CZT	PET	*P* value	CZT	PET	*P* value
Global	3.18 ± 0.95*	2.66 ± 0.92*	0.03	1.15 ± 0.31	1.02 ± 0.22	0.07 (NS)	2.84 ± 0.69	2.64 ± 0.81	0.32 (NS)
LAD	3.25 ± 0.97*	2.58 ± 0.92*	0.01	1.25 ± 0.32	1.04 ± 0.24	0.01	2.67 ± 0.77	2.52 ± 0.83	0.48 (NS)
LCx	3.33 ± 0.99*	2.72 ± 0.94*	0.02	1.26 ± 0.42	1.02 ± 0.25	0.01	2.80 ± 0.79	2.70 ± 0.83	0.64 (NS)
RCA	3.02 ± 1.14*	2.82 ± 1.18*	0.51 (NS)	1.09 ± 0.35	0.96 ± 0.25	0.1 (NS)	2.77 ± 0.70	2.99 ± 1.13	0.36 (NS)

*P < 0.001 vs. rest MBF

**Fig. 3:**
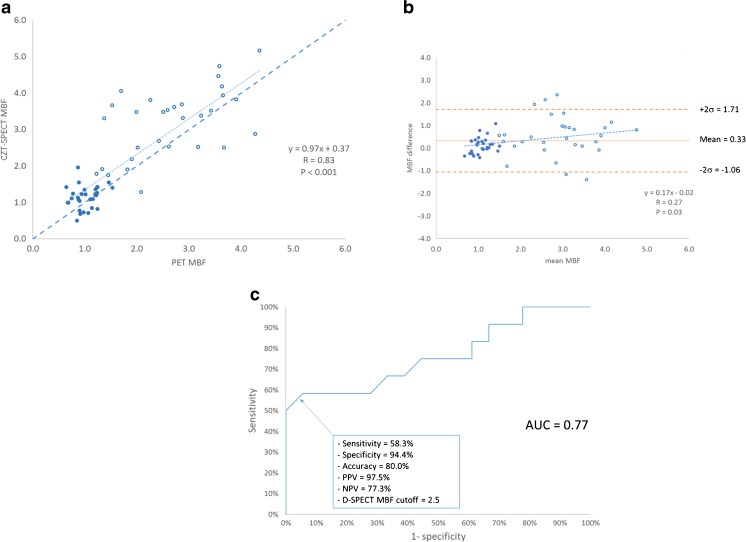
**a**. Correlation between global MBF (ml/min/g) by PET and by CZT-SPECT in 30 patients (rest: *full circle*; stress: *empty circles*). MBF estimates plotted against MBF measured by PET. The *dashed line* is the fit obtained from total least square regression. **b**. Bland-Altman plot showing the agreement between the two modalities in 30 patients for MBF (ml/min/g). **c**. ROC curve showing the sensitivity and specificity of CZT-SPECT for the diagnostic of abnormal stress PET MBF (< 2.5 ml/min/g)

As demonstrated in Fig. 3a and b, there was a good correlation between CZT-SPECT and PET for global MBF, with a mean difference between the two techniques of 0.33 (–1.06 to 1.71). On Bland-Altman plots, the difference between CZT-SPECT and PET varied slightly over a range of MBF. ROC curve analysis found a 2.5 (ml/min/g) cutoff for the diagnosis of abnormal stress MBF using CZT-SPECT with a sensitivity, specificity, accuracy, positive predictive value (PPV) and negative predictive value (NPV) of 58.3, 94.4, 80, 97.5 and 77.3%, respectively. The area under the curve (AUC) was 0.77 (Fig. 3c).

**Fig. 4:**
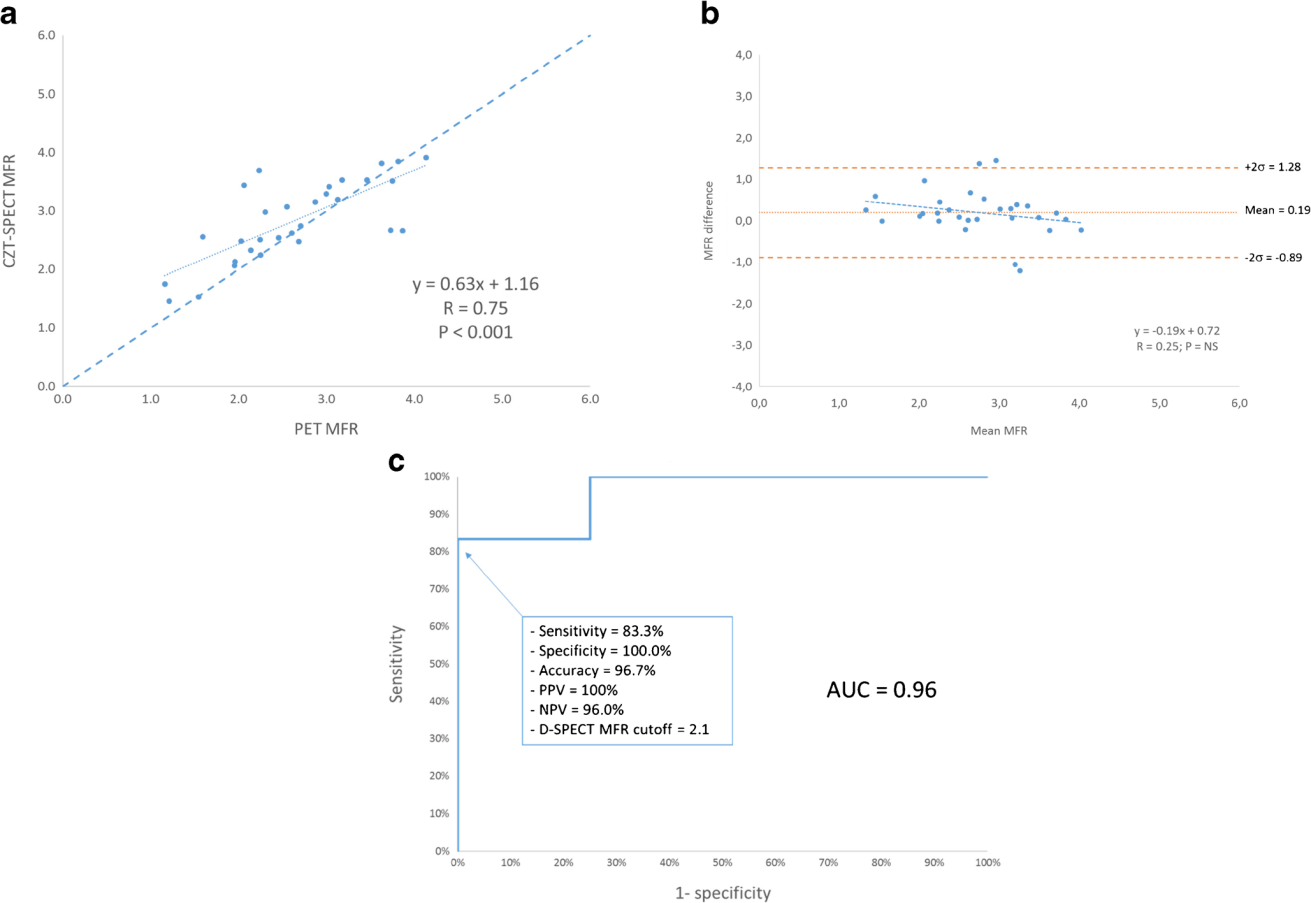
**a**. Correlation between PET and CZT-SPECT for global MFR in 30 patients. MFR estimates by CZT-SPECT plotted against MFR measured by PET. The *dashed line* is the fit obtained from total least square regression. **b**. Bland-Altman plots between PET and CZT-SPECT for global MFR in 30 patients. **c**. ROC curve showing the sensitivity and specificity of CZT-SPECT for the diagnostic of abnormal stress PET MFR (< 2)

There was also a significant correlation between CZT-SPECT and PET for global MFR, with a mean difference between the two techniques of 0.19 (–0.89 to 1.28; see Fig. 4a and b). In addition, the mean MFR did not vary between CZT-SPECT and PET. As depicted in Fig. 4c, the ROC curve found a cutoff of 2.1 for detection of abnormal MFR using CZT-SPECT, with a sensitivity, specificity, accuracy, PPV and NPV of 83.3, 100, 96.7, 100 and 96% respectively. The AUC was 0.96.

### Comparison of perfusion imaging vs. FFR

Despite the great number of vessels with significant stenosis, FFR was abnormal in only 12 coronary arteries (13% of the total number of coronary territories assessed). This low prevalence is likely to influence the predictive values of quantitative perfusion CZT-SPECT. However, there was a high concordance between MFR and FFR for both PET (kappa = 0.85) and CZT-SPECT (kappa = 0.81), as illustrated in Table 4. Discrepancies between FFR and CZT-SPECT and PET were observed in 17/90 and 13/90 vascular territories, respectively. The five territories with low FFR (≤0.8) and normal MFR (≥2.1) by CZT-SPECT corresponded to focal coronary lesions in five different patients. Most of these patients had an FFR value of 0.79 to 0.8, except one patient had an FFR of 0.66 on distal LAD. All of the remaining 12 patients with normal FFR (>0.8) and abnormal MFR (<2.1) by CZT-SPECT had more than three cardiovascular risk factors and five patients had diabetes. Nevertheless, MFR evaluated by CZT-SPECT and PET had similar diagnostic values for the detection of abnormal FFR (see Table 4).

**Table 4:** Concordance between FFR and MFR by PET and CZT in 90 artery territories in 30 patients

*N* = 90	FFR ≤ 0.8	FFR > 0.8
CZT-SPECT MFR < 2.1	7	12
CZT-SPECT MFR ≥ 2.1	5	66
PET MFR < 2	8	9
PET MFR ≥ 2	4	69
MFR vs. FFR	PET MFR < 2	CZT-SPECT < 2.1
Accuracy	86.7%	81.1%
Sensitivity (%)	66.7%	58.3%
Specificity (%)	88.5%	84.6%
Positive predictive value (%)	47.1%	36.8%
Negative predictive value (%)	94.5%	93%

**Fig. 5:**
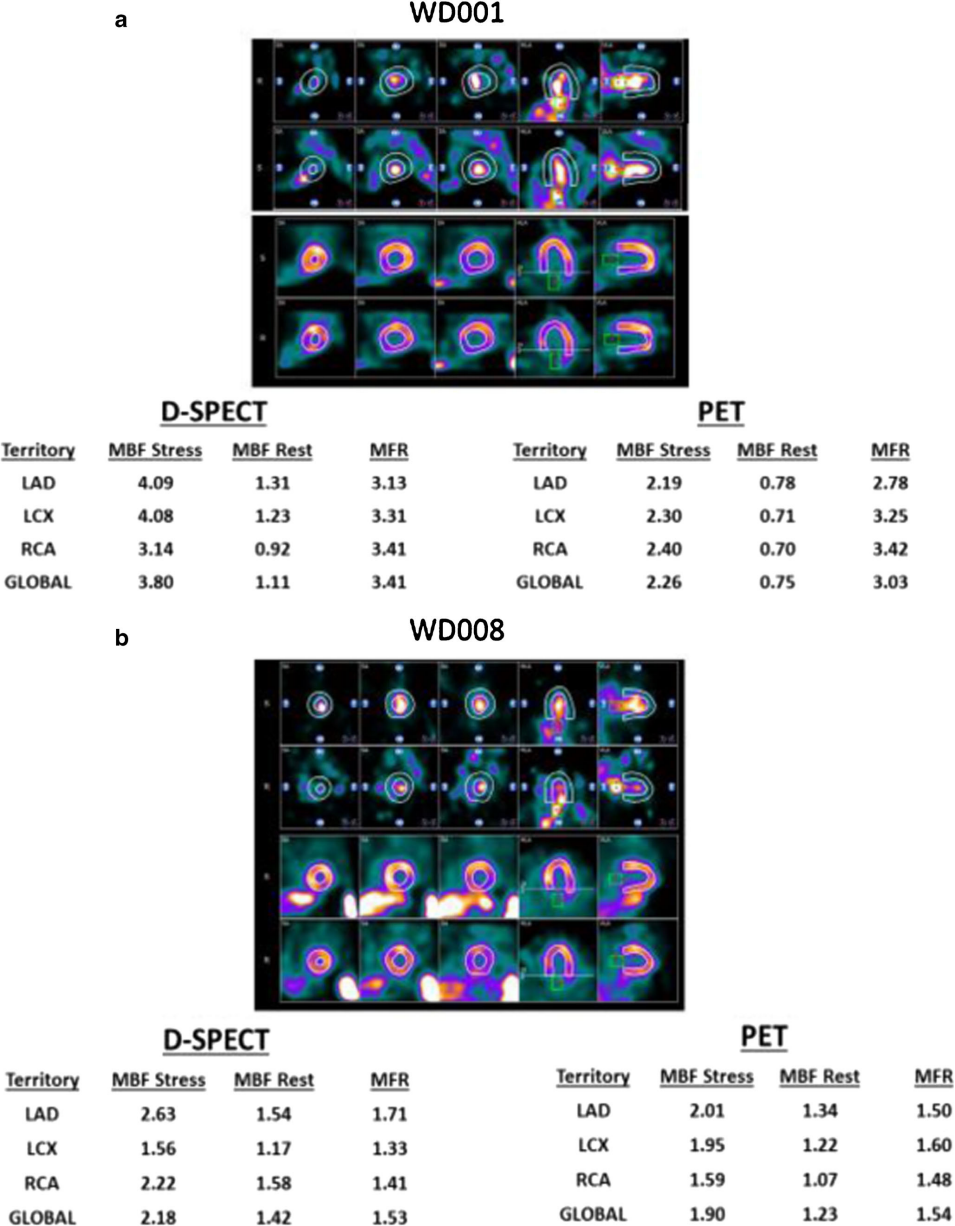
**a.** Patient W001 with normal FFR in the three coronary vessels: PET and CZT-SPECT both show normal MFR. MBF by PET and MBF by CZT-SPECT are also normal. Two stress and rest TACs are represented on the *right panel* during dynamic CZT-SPECT. Dynamic images show the bolus frame in the LA and LV followed by the LV myocardial activity frame. ROIs are placed on the LA for input function and on the global LV myocardium. **b.** Patient W008 with normal FFR in the three coronary vessels: PET and CZT-SPECT both show abnormal MFR (<2). Stress MBF by PET and MBF by CZT-SPECT are abnormal in LCx and RCA territories. Two stress and rest TACs are represented on the *right panel* during dynamic CZT-SPECT. Dynamic images show the bolus frame in the LA and LV followed by the LV myocardial activity frame. ROIs are placed on the LA for the input function and on global LV myocardium

Figure 5a shows a patient with normal FFR and normal global and regional MBF and MFR in CZT-SPECT and PET in all three vessels.

Figure 5b showed a patient with normal FFR but abnormal global MBF and MFR in CZT-SPECT and PET in all three vessels.

### Radiation dosimetry

The mean dose of ^99m^Tc-sestamibi was 294 MBq (169–443) at rest and 780 MBq (482–1229) at stress, leading to a total effective dose of 8.73 mSv (5.8–13.7) for dynamic SPECT [21]. The effective dose from the complete PET study (rest-stress H_2_^15^O and CT attenuation scan) was 3.1 mSv (2.6–4.2) [22, 23].

## Discussion

This is the first human study comparing MBF and MFR from dynamic ^99m^Tc-sestamibi CZT-SPECT with ^15^O–water PET and FFR, which are, respectively, considered gold standards for non-invasive MBF and MFR quantitative measurement [8, 20, 24], and invasive assessment of coronary stenosis severity [24, 25]. Our results demonstrate that quantification of MBF and MFR by dynamic ^99m^Tc-sestamibi CZT-SPECT is technically feasible with specific correction for the extraction fraction of ^99m^Tc-sestamibi [15]. While stress and rest MBF was significantly overestimated with CZT-SPECT compared to PET, MFR was similar with the two techniques. The key finding is that, nevertheless, values of CZT-SPECT MFR revealed an accuracy of 96.7% to assess abnormal MFR documented by ^15^O–water PET. In addition, CZT-SPECT MFR and MBF identified clinically significant CAD as defined by FFR with a diagnostic accuracy of 81.1%. However, MFR yielded much better diagnostic value than stress MBF (AUC: 0.96 vs. 0.77). SPECT is less accurate than PET for absolute quantification (MBF), but this limitation may be overcome by using the ratio of values from acquisitions performed in a very similar setting (MFR).

Previous studies exploring quantification of MBF with planar scintigraphy in humans [26–30] and with dynamic SPECT in experimental animals [31, 32] documented the limitations of conventional gamma cameras (slow rotation of the gantry and low count statistics). The high sensitivity, fast sampling and ROI-focused imaging capabilities of CZT-SPECT, as well as improvements in iterative reconstruction algorithms, enable dynamic cardiac SPECT studies, which can provide absolute quantitation of myocardial flow reserve [33, 34].

Using CZT-SPECT in a pig model of rest and transient occlusion at stress, Wells et al. [34] analyzed the data using a one-tissue compartment model with attenuation and scatter correction and reported a good correlation to microsphere flow values. Ben-Haim et al. [1] have assessed the technique in 95 patients with stable CAD. Factor analysis was used to generate right and left ventricular blood pool TAC which served as an input function in a two-compartment model, where MFR was calculated from K1 (^99m^Tc -sestamibi uptake) values at stress over rest and K2 (^99m^Tc-sestamibi washout) was set to zero. In this study, global and regional MFR showed good correlation with total perfusion deficit and with regional stress total perfusion deficit.

### CZT-SPECT vs. PET

Recently, Nkoulou et al. [3] reported lower MFR values with ^99m^Tc-tetrofosmin than typically seen with PET and ^13^N–ammonia, due to the lower MBF values observed by SPECT at high-flow conditions, while resting MBF values were highly comparable with PET. The underestimation of MBF with ^99m^Tc-tetrofosmin compared to ^13^N–ammonia is not only evidenced by a higher extraction fraction at resting conditions but also based on the fact that the given extraction fraction remains constant over a much larger range of MBF. Conversely, in the WATERDAY study, we observed increased global stress MBF values compared to ^15^O–water PET after correction of the ^99m^Tc-sestamibi extraction fraction [15], while resting MBF values were highly comparable with ^15^O–water PET. The difference between the two techniques increased for greater flow values, likely due to the absence of AC in CZT-SPECT. The plot shows that the agreement between SPECT and PET is rather good when MBF is <2 ml/min/g (resting conditions), and decreases for greater flow values (vasodilation). One of the reasons can be the difficulty to distinguish the myocardial edges because of photon attenuation, leading to a possible myocardial ROI positioning error inducing some fluctuations in MBF calculation. In addition, the absence of AC results in increasing the height of the input function curve, causing a distortion of the input function and hence a significant overestimation in the calculated MBF values [23].

Regional analysis demonstrated an increased stress and rest MBF in LAD and LCx using CZT-SPECT compared with PET. CZT-SPECT provided global and regional MFR similar to PET and yielded high diagnostic values for the detection of impaired PET flow reserve with high PPVs and NPVs (respectively, 100% and 96%) in our population.

Despite the limited sample size, the strength of these results lies in the fact that each patient served as his own control and the cut-off for abnormal perfusion reserve was a widely accepted PET MFR threshold (<2) [33–35]. ^15^O–water PET has high reproducibility and agreement between software packages [7], but shares several limitations with ^99m^Tc-sestamibi CZT-SPECT, including potential problems with bolus delivery, patient motion and suboptimal hyperaemia. The agreement between software packages for dynamic ^99m^Tc-sestamibi tracer using CZT cameras should be assessed in further studies. MFR reflects CAD beyond the epicardial section of the coronary tree involving microcirculation and endothelial dysfunction. PET studies showed an increased sensitivity in detecting CAD using global MFR as an adjunct to perfusion analysis [35]. Therefore, it is of paramount importance to develop dynamic SPECT imaging that could benefit a wider cardiac patient population. Our results demonstrated high agreement of dynamic CZT-SPECT with PET for the identification of abnormal global and regional MFR. However, per-segment (17 segment model) MFR analysis was not performed as segmental counts statistics were not sufficient enough to draw high-quality TACs compatible with reliable measurements.

The absence of AC may have an impact on flow quantification with the CZT camera. To quantify MBF, we have adjusted the input function ROI inside the LA in order to avoid crosstalk between myocardial counts and bolus ROI that is mainly due to motion (respiration, heartbeat, etc.). However, the LA is often deeper inside the body and will be more affected by the attenuation (~25% attenuation for 2 cm at 140 keV).

### CZT-SPECT vs. FFR

FFR was initially introduced as an invasive surrogate marker of relative severity of myocardial ischemia, and validated by comparison with PET-derived relative coronary flow reserve [36]. PCI has shown improved prognosis in patients with abnormal FFR, using a cut-off value between 0.75 [37] and 0.80 [10, 11]. Between these two limits, there is a substantial gray zone with a certainty of 99% that a given lesion is responsible for ischemia. Ironically, FFR was recently proposed as a reference to validate novel non-invasive flow-based functional assessment by PET [2, 20]. A key finding of our study is that a 2.1 cut-off value for CZT-SPECT MFR predicted an abnormal FFR (<0.80) with an accuracy of 81.1% and an NPV of 93%, comparable to ^15^O–water PET.

In our study, some of the false positives (12 patients) were likely due to the presence of microvascular dysfunction in patients with diabetes or with more than 3 cardio-vascular risks, highlighted as “slow flow” during ICA with a normal FFR. FFR is primarily focused on identifying a pressure gradient across a stenosis, but cannot directly assess microvascular resistance or flow. However, high specificity (84.6% vs. FFR) was an important finding using CZT-SPECT, thus supporting the use of this new technique to guide referrals for ICA or as an optional “add-on” investigation after ICA/FFR when the functional severity of stenosis is uncertain or when FFR is contraindicated (suboccluded artery, asthma). Some of the false negatives (five patients) were due to focal or unique lesions on the main coronary arteries identified by ICA, with a FFR less than 0.8. However, in most of these cases (4/5 patients) the FFR was within the gray zone, between 0.79 and 0.8 [19, 20]. In contrast, global and even regional MBF and MFR were in the normal range.

Due to a poor distinction between epicardial stenosis and microvascular disease, it is quite challenging to directly correlate MBF with a specific epicardial lesion. Further, the widely standardized segment model [38] is frequently inaccurate in the prediction of a diseased coronary branch; an area at risk can be mixed with adjacent normal areas or truncated by the crude segmentation.

### MFR -CZT and MFR-PET vs. FFR in vessel territories

For LAD and LCx vessels, when FFR was less than 0.8, MFR (< 2) by PET had better accuracy, specificity, PPV and NPV than MFR (2.1) by CZT. However, for RCA when FFR was less than 0.8, the accuracy was 86.7%, the sensitivity was 66.7%, the specificity was 88.9%, the PPV was 40% and the NPV was 96% for both MFR modalities. The concordance between FFR and MFR-PET was slightly better than MFR-CZT in all 90 territories with a kappa coefficient as 0.85 and 0.81, respectively. The difference in the regional MBF and MFR values could be attributed to the use of attenuation correction (AC) in PET imaging.

### Study limitations

It would be informative to perform myocardial perfusion imaging (MPI) to obtain semiquantitative assessment of perfusion analysis (expressed as total perfusion deficit or summed scores), to show potential additional diagnostic value of flow over uptake, or of a combination of both; but such conventional acquisitions have not been finally done in this trial. Although we demonstrated the capability of CZT-SPECT MBF quantification to be close to PET quantification, the number of investigational subjects and, in particular, those with severe stenosis was still small. Future studies should enroll a larger number of subjects, and ideally utilize both attenuation and scatter correction, neither of which was performed in this study. Variability due to methodology arises from the myocardial radioactive tracer distribution (^99m^Tc-sestamibi vs. ^15^O–water), reconstruction algorithms, and flow model (^99m^Tc-sestamibi vs. ^15^O–water). However, the flow model for ^99m^Tc-sestamibi used in this study has been previously validated in animals [15].

## Conclusion

Quantification of MBF and MFR values by dynamic CZT-SPECT MPI with ^99m^Tc-sestamibi was technically feasible. Hyperemic and rest values were significantly higher than from ^15^O–water PET, but resulted in similar MFR. Finally, the high diagnostic value for detecting impaired MFR and abnormal FFR supports use of this new technique as an optional “add-on” investigation after ICA when the functional severity of stenosis is uncertain or FFR is contraindicated.

## Abbreviations

BP: Blood pressure
CAD: Coronary artery disease
CT: Computed tomography
CZT: Cadmium-zinc-telluride
DLP: Dose-length product
EF: Extraction fraction
FFR: Fractional flow reserve
HR: Heart rate
ICA: Invasive coronary angiography
i.v.: Intravenous
LAD: Left anterior descending artery
LA: Left atrium
LV: Left ventricle
LCx: Left circumflex
MBF: Myocardial blood flow
MFR: Myocardial flow reserve
MPI: Myocardial perfusion imaging
PET: Positron emission tomography
RCA: Right coronary artery
ROI: Region of interest
SPECT: Single-photon emission computed tomography
TAC: Time activity curves
